# Modulation of Cell Death Pathways for Cellular Protection and Anti-Tumoral Activity: The Role of *Thymus* spp. Extracts and Their Bioactive Molecules

**DOI:** 10.3390/ijms24021691

**Published:** 2023-01-14

**Authors:** Carlos Martins-Gomes, Fernando M. Nunes, Amélia M. Silva

**Affiliations:** 1 Centre for Research and Technology of Agro-Environmental and Biological Sciences (CITAB), Cell Biology and Biochemistry Lab, University of Trás-os-Montes and Alto Douro (UTAD), Quinta de Prados, 5001-801 Vila Real, Portugal; 2 Chemistry Research Centre-Vila Real (CQ-VR), Food and Wine Chemistry Lab, UTAD Quinta de Prados, 5001-801 Vila Real, Portugal; 3 Department of Chemistry, School of Life Sciences and Environment, UTAD, 5001-801 Vila Real, Portugal; 4 Department of Biology and Environment, School of Life Sciences and Environment, UTAD, 5001-801 Vila Real, Portugal

**Keywords:** apoptosis, autophagy, necrosis, cell cycle, cell death, cell survival, *Thymus* genus, phytochemicals, phenolic acids, flavonoids, pentacyclic triterpenoids

## Abstract

Natural products used for their health-promoting properties have accompanied the evolution of humanity. Nowadays, as an effort to scientifically validate the health-promoting effects described by traditional medicine, an ever-growing number of bioactivities are being described for natural products and the phytochemicals that constitute them. Among them, medicinal plants and more specifically the *Thymus* genus spp., arise as products already present in the diet and with high acceptance, that are a source of phytochemicals with high pharmacological value. Phenolic acids, flavonoid glycoside derivatives, and terpenoids from *Thymus* spp. have been described for their ability to modulate cell death and survival pathways, much-valued bioactivities in the pharmaceutical industry, that continually sought-after new formulations to prevent undesired cell death or to control cell proliferation. Among these, wound treatment, protection from endogenous/exogenous toxic molecules, or the induction of selective cell death, such as the search for new anti-tumoral agents, arise as main objectives. This review summarizes and discusses studies on *Thymus* spp., as well as on compounds present in their extracts, with regard to their health-promoting effects involving the modulation of cell death or survival signaling pathways. In addition, studies regarding the main bioactive molecules and their cellular molecular targets were also reviewed. Concerning cell survival and proliferation, *Thymus* spp. present themselves as an option for new formulations designed for wound healing and protection against chemicals-induced toxicity. However, *Thymus* spp. extracts and some of their compounds regulate cell death, presenting anti-tumoral activity. Therefore *Thymus* spp. is a rich source of compounds with nutraceutical and pharmaceutical value.

## 1. Introduction

When considering cell death induction, oncological treatment is one of the main areas where science wishes to regulate the selective induction of cell death. Irrefutably, paclitaxel (trademark Taxol^®^), is a crucial intervenient and the most well-known drug used in chemotherapy [1]. This terpenoid (tetracyclic diterpenoid) is produced mainly by *Taxus* spp., and after the discovery of its molecular structure in 1971, since the initial clinical trials and development of a chemical synthesis protocol, paclitaxel has become an undeniable example of the pharmacological potential of phytochemicals [1]. On the other hand, pharmaceutical products that treat ailments or promote cell proliferation are also highly sought after. In this field, natural products have been shown to be sources of phytochemicals with wound-healing properties [2,3], which, combined with their anti-inflammatory activity [4], have been shown to highly contribute to assuring cell proliferation and faster wound closure. In addition, phytochemicals may play a significant role in, for example, neuroprotection [5] and intestinal homeostasis [6], where preventing undesired cell death caused by internal or external insults is of great interest.

There is also a middle ground between anti-proliferative activity, desired to fight tumoral cells, and the proliferative activity targeting normal tissue, which is the chemopreventive activity and/or chemotherapy drug adjuvants. The first is due to the activity of phytochemicals in preventing tumoral development, but also to their protective effect towards non-tumoral cells when exposed to xenobiotics, such as chemotherapy drugs [7]. The adjuvant activity is mainly due to phytochemicals’ potential to reverse multidrug resistance or to favor drugs reaching the target cell by facilitating permeability, thus increasing the effectiveness of chemotherapy drugs [8].

Although presenting a high impact in the modern pharmaceutical industry, drugs originated exclusively by chemical synthesis still represented a smaller fraction of the new pharmaceutical options available (36% between 1981–2010), while a major share was represented by small molecules based on natural products [9]. Despite presenting limitations related to nomenclature issues, the variability in the intrinsic phytochemical composition, plus that induced by edaphoclimatic conditions or low yields, were aspects that the pharmaceutical industry aimed to overcome with chemical synthesis, however, plants can produce an immense number of chemical structures and variations of them [10,11]. Thus plants produce a high number of different compounds, that may present pharmaceutical potential but whose chemical synthesis would be very complex [10], with high costs. Considering anti-cancer drugs, from about 1940’s to 2010, 175 small molecules were identified as drugs with anti-tumoral activity, of which 48.6% represent natural products, their derivatives, or synthetic products with natural products as pharmacophores. Among them, we can find drugs commonly used in chemotherapy such as paclitaxel, vinblastine or irinotecan, and others [12]. Nevertheless, the pharmaceutical relevance of phytochemicals extends to more uses than chemotherapy, with a high impact among antimicrobials and anti-diabetic drugs for example [12]. Cell proliferation induced by natural products is understudied when compared to anti-proliferative activity aimed at cancer treatment. Often used in traditional medicine, natural products are generally regarded as safe [13], and thus most studies are directed towards the study of bioactivities such as antioxidant, antimicrobial or anti-inflammatory, with raising awareness for the evaluation of safety profiles. Fewer studies are found reporting proliferative activity. For example for osteoporosis treatment purposes, natural products used in traditional Chinese medicine have been shown to induce osteoblasts proliferation and differentiation [14].

According to the most recent update (December 2022) of the International Union for Conservation of Nature (IUCN), more than 424,000 plant species can be currently estimated [15], from which only approximately 60,000 species have been analyzed for the pharmaceutical value of their constituents, and originated 135 drugs [9]. Thus, despite the already scientifically validated potential, the use of natural products in the pharmaceutical industry is yet far from its full potential, being expected that with the screening of understudied species, new candidates for drug leads will appear that aim at protecting, proliferating and killing of target cells.

Among the sources of phytochemicals, medicinal and aromatic plants are highly praised for their wide range of bioactivities. With a long history of use, from ancient traditional medicine to the current date, these health-promoting effects are being consolidated by the scientific community, to correlate biological effects with the molecular structure of phytochemicals to identify cellular targets. These efforts are driven by the need for a new treatment or disease prevention options for an ever-increasing population [16]. In this review, we provide a summary of findings regarding the ability of *Thymus* spp. Extracts in modulating cell survival and death pathways, as well as the role of their main phytochemicals in those processes. We emphasize the role of phenolic acids, such as caffeic (CA), rosmarinic (RA), or salvianolic acids (Sas), glycoside derivatives of luteolin, apigenin, quercetin or eriodictyol, and the effect of pentacyclic triterpenoids, all commonly found in *Thymus* genus, as will be discussed below.

## 2. Signaling Pathways in Cell Survival and Cell Death

Cell death regulation is a very intricate process, that is designed to eliminate undesired, damaged, or problematic cells based on different stimuli and for different purposes [17,18]. These mechanisms are often split into two major types of cell death, the ones caused by physical stimuli, like trauma, that result in necrosis. The second type comprises the programmed cell death pathways, which are dependent on signaling cascades that interconnect and develop cellular responses that culminate in the elimination of cellular debris [18,19]. In recent years it was described that necrosis can be regulated and mediated by signaling pathways, in which the necroptosis process is the one that is better described, but ferroptosis and pyroptosis have also been described [17,19]. Nevertheless, the initial signals and already defined hallmarks of each process can be used to distinguish apoptosis and necrosis. While apoptosis implies caspases activation and a strict order of events resulting in phagocytosis of debris aiming minimal inflammatory process (although apoptosis can, occasionally, induce the immune system), necrotic cell death was regarded as passive, caused by trauma-induced membrane rupture, or cell swelling followed by membrane rupture, leaking of intracellular components and then triggering inflammation [20].

### 2.1. Regulated Necrosis

**Figure 1:**
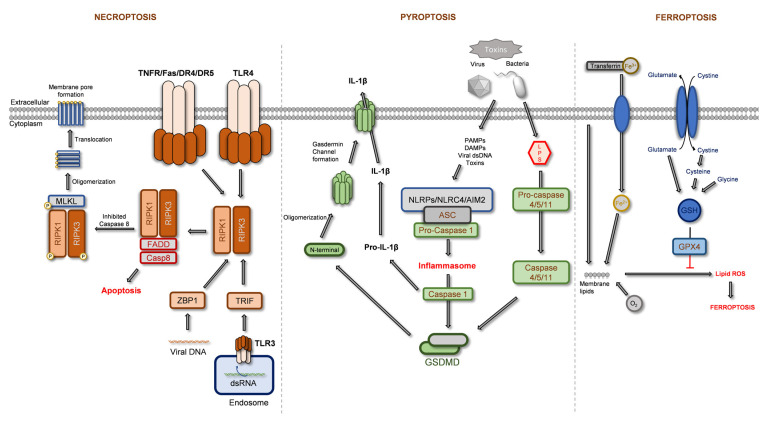
Schematization of signaling pathways of regulated forms of necrosis: necroptosis, pyroptosis, and ferroptosis. Adapted from [20,21,22,23,24,25,26,27,28,29,30], and from illustrations courtesy of Cell Signaling Technology [31], Inc.. The red arrow means inhibition; the grey arrow means inducer. Abbreviations: AIM2, Interferon-inducible protein AIM2; ASC, apoptosis-associated speck-like protein containing a CARD; Casp8, caspase-8; DAMPs, damage-associated molecular patterns; FADD, Fas-associated death domain; IL-1β, interleukin 1β, LPS, lipopolysaccharide; NLRC4, NLR family CARD domain-containing protein 4; NLRPs, NLR family pyrin domain containing; PAMPs, pathogen-associated molecular patterns; TRIF, TIR-domain-containing adapter-inducing interferon-β; All other abbreviations are mentioned in the text.

The regulated forms of necrotic cell death comprise necroptosis, pyroptosis, and ferroptosis pathways. The signaling pathways of these processes are illustrated in Figure 1. As described below, these regulated necrotic processes vary on the signaling pathways used, culminating in the loss of membrane integrity and leakage of intracellular constituents, both hallmarks of necrosis. In addition, while being studied in less extent when compared to apoptosis, new molecular markers, as well as new inhibitors of these pathways, have been reported, with high significance in current and future studies, as described below.

#### 2.1.1. Necroptosis

Necroptosis presents morphological hallmarks typical of necrotic death: no chromatin condensation, swelling, and rounding of the cell and dilated organelles; but just as other regulated cell death types, this is driven by signaling pathways. Necroptosis has already been correlated with chemotherapy drugs-induced cytotoxicity, and identified in pathologies such as myocardial infarction psoriasis, cirrhosis, pancreatitis, neurodegenerative diseases, or ischemic injury [17,20]. This pathway is highly dependent on RIPK1 (receptor-interacting serine/threonine-protein kinase 1) and RIPK3 (receptor-interacting serine/threonine-protein kinase 3), which have been used as markers of necroptosis [17].

Considering the signaling pathway, necroptosis signaling is mediated through stimuli induced in tool-like receptors 3 and 4 (TLR3/TLR4), tumor necrosis factor receptor 1 (TNFR1), Fas receptor (also called Fas, CD95 or apoptosis antigen 1), death receptors 4 and 5 (DR4/DR5) or Z-DNA-binding protein-1 (ZBP1) [19,20]. In this last one, the stimuli originated from nucleic acids of pathogens, such as a virus.

**Figure 2:**
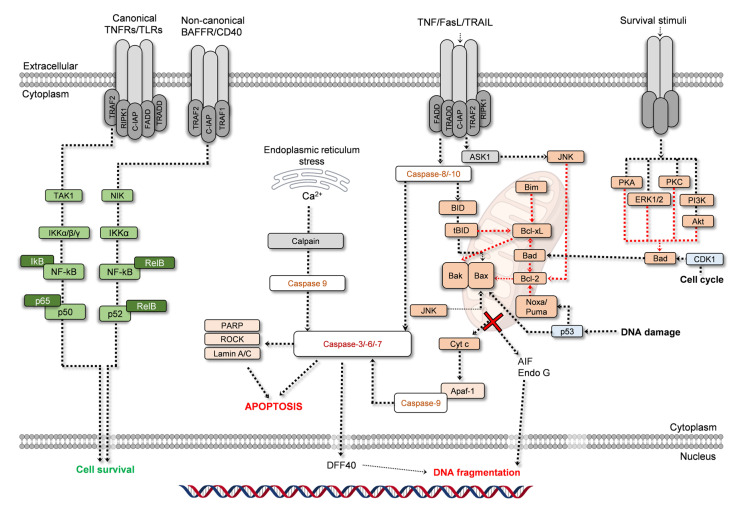
Schematization of main intervenients participating in key signaling pathways in cell survival and apoptosis processes. Adapted from [37,38,39,40,41,42,43,44], and from illustrations courtesy of Cell Signaling Technology, Inc. [45,46,47]. The red arrow means inhibition; the black arrow means inducer. Abbreviations: ASK1, apoptosis signal-regulating kinase 1; c-IAP, cellular inhibitor of apoptosis; FADD, Fas-associated death domain; TRADD, tumor necrosis factor receptor type 1-associated death domain; TRAF, TNF receptor-associated factor. All other abbreviations are mentioned in the text.

For all the receptors mentioned above, RIPK3 recruitment is required, while RIPK1 recruitment is required for Fas, TNFR1, and DR4/5, where it binds to RIPK3. Upon this stage, RIPK3 is phosphorylated, to form the necrosome, which then activates MLKL (mixed lineage kinase domain-like pseudokinase), which triggers a change in conformation and migration to the cell membrane, inducing membrane permeabilization, leakage of intracellular content and inflammation [19,20]. Being a regulated process, necroptosis maintains a strict connection with apoptosis and cell proliferation. Caspase-8, for example, is a regulator of necroptosis. When increased, the apoptotic pathway is favored, as caspase-8 is part of the apoptotic signaling cascade, but also because of its inhibitory effect over RIPK1 and RIPK3. On the other hand, RIPK1 is necessary for survival pathways activation, as is the case of JNK (c-Jun N-terminal kinases), MAPK/ERK (mitogen-activated protein kinases/extracellular signal-regulated kinases), and NF-kB (nuclear factor kappa-light-chain-enhancer of activated B cells) [17,18,19,20]. NF-kB pathway, for example, may be activated by growth factors, cytokines, or downstream targets originating in other signaling cascades. It presents two routes, canonical and non-canonical (Figure 2; left side; green). NF-kB pathway is initiated at TNFRs, TLRs, BAFFR (B-cell activating factor receptor), or CD40 (cluster of differentiation 40), depending on the route. In the canonical pathway, TAK1 (transforming growth factor (TGF) β-activated kinase 1), currently known as MAP3K7 (mitogen-activated protein kinase kinase kinase 7), activates IKKα, IKKβ, and IKKγ, leading to the removal of IKB, thus leaving the subunits p65 and p50 free to translocate to the nucleus. A similar process is conducted in the non-canonical pathway, but only IKKα is activated, by NIK (mitogen-activated protein kinase 14/NF-kB-inducing kinase), leading to the activation of the complex RelB-p52 [32,33]. In both pathways occurs the translocation of the subunit to the nucleus, triggering various responses, namely toward cell survival. NF-kB can suppress apoptosis [32].

A highlight in necroptosis is the fact that phosphatidylserine (only present in the internal leaflet of the membrane in healthy cells) externalization can also occur, a change in the cell’s membrane that was thought to be a marker of apoptosis. Annexin V-FITC is a very common probe used to detect apoptosis, but in fact, other forms of regulated cell death may induce phosphatidylserine externalization [34]. This effect is also valid for pyroptosis, thus annexin V used alone cannot assure the occurrence of apoptosis [35].

In addition to necroptosis, also pyroptosis and ferroptosis have been described as regulated forms of necrosis.

#### 2.1.2. Pyroptosis

Pyroptosis is defined as an inflammatory form of necrosis, based on the activity of inflammatory proteases and the formation of an inflammasome. The main intervenients in pyroptosis are gasdermin and the NOD-like receptors (NLR), in a process illustrated in Figure 1. Induced by toxins, or pathogen infections (e.g., viruses and bacteria), pyroptosis activation may initiate in the LPS-dependent activation of caspase-4, -5, and -11, or through the formation of the inflammasome, involving NLRs, NLRC4 (NLR family CARD domain-containing protein 4) or AIM2 (interferon-inducible protein AIM2), ASC (apoptosis-associated speck-like protein containing a CARD), and pro-caspase 1, leading to caspase 1 activation. Both pathways culminate in gasdermin cleavage. Gasdermin’s *N*-terminal portion undergoes oligomerization, followed by a translocation to the plasmatic membrane, forming the gasdermin pores that allow leakage of pro-inflammatory factors such as IL-1β [23,24,25,35]. Interestingly, this process implies the involvement of caspase-1, -3, -4, -5, -6, -8, -9, and -11, maintaining some similarities with apoptosis in which caspase-3, -6, -8, and -9 are also activated [25,35]. This pathway is associated with tumor development, being a druggable target that is increasingly under study [25].

#### 2.1.3. Ferroptosis

Ferroptosis is an iron-dependent form of programmed cell death, which differs from necroptosis and pyroptosis in its origin, being triggered by inhibition in glutathione synthesis or inhibition of GPX4 (glutathione-dependent antioxidant enzyme glutathione peroxidase 4), constituting the intrinsic pathway. On the other hand, in the extrinsic pathway, the main triggers are the inhibition of transmembrane transporter xCT (cystine/glutamate transporter; exchanges intracellular glutamate for extracellular cysteine) or modulation of iron transporters (e.g., transferrin) [28,29,30]. Overall, the pathway leads to the formation of lipid reactive oxygen species in an iron-mediated process, inducing lipid peroxidation, and affecting the membrane’s integrity. Ferrostatin-1 is a ferroptosis inhibitor commonly used to assess ferroptosis [28,29,30].

Both pyroptosis and ferroptosis pathways are associated with tumor development, being a druggable target that is increasingly under study [18,21,23].

### 2.2. Apoptosis

The apoptotic process is the most studied form of programmed cell death, and the most addressed when studying the proliferative/anti-proliferative activities of natural products. For this reason, it is also the one with the highest number of markers, allowing it to be easily detected. Apoptosis presents various morphological hallmarks that allow it to be distinguished from other processes. A cell undergoing apoptosis starts to detach from the surrounding cells, and the cytoplasm condenses causing the cell to shrink. At the nucleus, the DNA initially condensates, and then fragments. In the later stages, membrane blebs are formed, and then the cell is fragmented into apoptotic bodies that are discarded by phagocytosis. However, morphological changes are not sufficient to determine if apoptosis is occurring [36]. For this reason, specific targets of the apoptotic cascade are used in studies aiming to assess which pathway is active. For apoptosis, there are a few intervenients that make a presence in most studies, and their role in the apoptotic process is illustrated in Figure 2.

Firstly, apoptosis is divided into two distinct, but interconnected, routes, the intrinsic and the extrinsic pathways. The first is described as being mitochondria-dependent, where many intracellular stimuli such as DNA damage, uncontrolled division, or oxidative stress can induce this pathway. The extrinsic pathway originates from extracellular stimuli, arising from the activation of cell-surface death receptors by ligands such as TNF-α, FasL (Fas ligand), or TRAIL (TNF-related apoptosis-inducing ligand). Activation of the extrinsic pathway may also induce the intrinsic pathway. Regardless of the pathway, both culminate in caspase -3, -6, and -7 activation (Figure 2). In fact, caspases are also known as hallmarks of apoptosis, which is often described as a caspase-dependent pathway [48].

The Caspases family comprises cysteine-dependent endoproteases mostly known for their activity in apoptosis. Caspases are also classified based on their functions: caspase-1, -4, -5, and -11 are classified as inflammatory; caspase-3, -6, and -7 are executioner caspases in apoptosis; caspase -8, -9, -10 are initiators in apoptosis; caspase-2 is associated with cell cycle regulation; caspase-14 is involved in cell differentiation [49,50]. Caspase-12 is not expressed in a large portion of the human population. Originally was thought to mediate apoptosis induced by stress in the endoplasmic reticulum, but recent findings point out that caspase-12 may not play a significant role in this pathway and its role in apoptosis is yet to be fully unveiled [49]. Caspase-14 participates in epidermis cornification and protection, and caspase-16 functions are still unclear [50]. Thus, it is clear that the caspases family plays a role in various essential processes, and in special are involved in various forms of regulated cell death [50].

A second highly relevant group of proteins that participate in the apoptotic process are comprised of the Bcl-2 (B-cell lymphoma 2) family. As pro-apoptotic proteins: effector proteins group where we can find Bak (Bcl-2 homologous antagonist/killer) and Bax (BCL-2-associated x protein; bcl-2-like protein 4); a second group includes BID (BH3 interacting-domain death agonist) and Bim (Bcl-2-like protein 4/Bcl-2 interacting mediator of cell death) and are known as direct activators BH3-only proteins; Noxa (phorbol-12-myristate-13-acetate-induced protein 1), Puma (p53 upregulated modulator of apoptosis) and BAD (Bcl-2 associated agonist of cell death) are sensitizers/de-repressors BH3-only proteins. Among the anti-apoptotic members, the highlight goes to Bcl-2 and Bcl-xL (Bcl-2-like protein 1), a classification based on their structure [30,31,44]. All these proteins play a significant role in apoptosis.

As seen in Figure 2, in a simplified scheme, Noxa, Puma, and Bad can inhibit the activity of anti-apoptotic proteins Bcl-2 and Bcl-xL. Thus, the anti-apoptotic activity over Bax and Bak is terminated, initiating the process of mitochondrial-outer membrane permeabilization. Noxa and Puma activity may be induced by p53, while Bad action can be driven by cell cycle modulation, or the modulation of, for example, PI3K (phosphoinositide 3-kinase)/Akt (protein kinase B) signaling pathway, that once inhibited stops sending survival stimuli. Bim and BID also inhibit the anti-apoptotic proteins. Other pathways, such as JNK, can also induce Bax activity. The oligomerization of Bak and Bax is a critical step in mitochondrial outer membrane permeabilization, leading to cytochrome c (cyt c) release into the cytoplasm, a point from which the apoptosome forms, a structure that contains cyt c, APAF-1 (apoptotic protease-activating factor-1), deoxyadenosine triphosphate and pro-caspase-9 [37,38,39,40,41,51,52,53,54]. Once pro-caspase-9 is activated into caspase-9, the next step is the activation of caspase-3 and caspase-7. The sequence of events described until here constitutes the intrinsic pathway of apoptosis (Figure 2). The extrinsic pathway differs from the intrinsic pathway in all the upstream events until reaching this point, as caspase-3, and caspase-7 activation is mediated by caspase-8 and -10. Activated caspase-8, and -10, can also activate BID, inducing the intrinsic pathway (Figure 2), and interconnecting the two pathways.

The final steps in apoptosis involve PARP (poly-(ADP-ribose) polymerase) cleavage and the translocation of factors such as DFF40 (DNA fragmentation factor 40; DNase activated by caspase), AIF (apoptosis-inducing factor) or Endo G (endonuclease G) into the nucleus, inducing DNA fragmentation. All these signaling steps are accompanied by the morphological hallmarks stated above, for example, ROCK protein (Rho-associated protein kinase; Figure 2) is involved in the cell membrane blebbing characteristic of apoptosis [37,38,39,40,41,42,43,44,51,52,53,54,55,56].

As will be discussed later in this review, natural products, and their phytochemicals act over anti-apoptotic proteins controlling their activity, but also modulate pathways, such as ERK/PKC/PI3K/Akt signaling axis, controlling survival stimuli and thus modulating apoptosis. Together with Bcl-2, Bcl-xL, PARP, Bax, Bad, and caspases, these are common markers used in studies reporting the modulation of cell death by phytochemicals.

### 2.3. Autophagy

**Figure 3:**
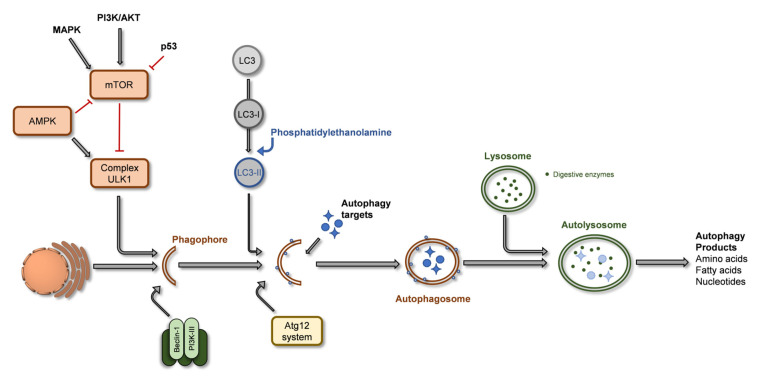
Schematization of main intervenients participating in autophagy signaling pathways. Adapted from [61,62,63,64], and from illustrations courtesy of Cell Signaling Technology, Inc. [65]. Red arrows mean inhibition; grey arrows mean induction. Abbreviations: ULK1, Unc-51 like autophagy activating kinase. All other abbreviations are mentioned in the text.

Various diseases such as Crohn’s, fatty liver, cardiomyopathy, or diabetes are linked to malfunctions in the autophagic process, which is an essential homeostatic process [57]. The autophagic process provides a tool to eliminate cellular waste, allows the turnover of organelles and other intracellular components, and can maintain a cell’s metabolism under nutrient stress. In addition, autophagy’s relevance to preventing tumorigenesis is also recognized [58,59]. The autophagic process is illustrated in Figure 3, involving the formation of the phagophore, a double membrane vesicle formed from the endoplasmic reticulum (ER)/Golgi complex membrane that harvests the components to recycle, becoming the autophagosome, that maturate and which then fuses with lysosomes to produce autolysosomes, whose enzymatic content digests proteins, organelles or other intracellular constituents [59,60]. During this process, a specific protein, LC3 (microtubule-associated proteins 1A/1B light chain 3B), is recruited to the autophagosome and is a key in the autophagic process [60].

Autophagy detection is mainly performed through the expression of LC3 protein [66]. This protein is originally synthesized as pro-LC3, and presents a cytosolic form, LC3-I, usually defined as a soluble form or unlipidated form. Conversion of LC-I into LC3-II occurs when it conjugates with phosphatidylethanolamine (lipidated form) during the autophagosome formation, steps catalyzed by autophagic related (Atg) proteins family [57,66]. LC3-II function comprises the selection of targets to engulf and recycle and the hemifusion [59]. Thus, LC3-II expression is increased during autophagy, as the autophagosome forms, yet its degradation is also initiated during the autophagosome-lysosome fusion process. In this step LC3-II is recycled, being converted to LC3-I. For this reason, although LC3-II is indicative of autophagosome formation in relation to autophagy, its expression may not exactly serve as a quantitative measure of the autophagic activity but the relation LC3-II/LC3-I is a better marker. A second marker for autophagy is p62, which binds to LC3, and has been shown to increase in case of autophagy inhibition [57,66]. During the phagophore formation phase, the protein beclin-1 plays a critical role by forming a complex with class III phosphatidyl-inositide 3-kinase, Vps34, a PI3-kinase in the endoplasmic reticulum, triggering nucleation and formation of the autophagosome. Low expression levels of Beclin-1 (or its absence) are related to the prevalence of specific types of cancer, such as breast cancer, hepatic cancer, or lymphoma [59]. Beclin-1 levels determine whether cells undergo autophagy or apoptosis. Beclin-1 also interacts with the anti-apoptotic protein Bcl-2, forming a Bcl-2:Beclin 1 complex, inhibiting autophagy by preventing beclin-1 from forming the autophagosome. This is one of the connections between autophagy and apoptosis, as both can potentiate or decrease the chances of the other. For example, p62 is an intervenient in caspase-8 activation but also in the selection of autophagy targets [67]. NF-kB pathway promotes autophagy but prevents apoptosis. P53 can upregulate and downregulate autophagy, and it is also known that caspase-3 downregulates autophagy at later stages of apoptosis through beclin-1 cleavage [67]. Due to the complex relationship between both processes, the interconnection of the various pathways is not yet completely clear, and it is not consensual which process activates/inhibits the other. Although these are pathways commonly modulated by natural compounds (such as phytochemicals), these are not the only types of cell death, as other types like parthanatos (a cell death process that involves DNA damage-responsive enzymes, poly(ADP-ribose) polymerase (PARP) proteins, in particular, PARP1 that is independent of caspases), paraptosis (a growth receptor hyper-activation dependent cell death process), oncosis (ischemic cell death), or mitoptosis (cell death promoted by loss of mitochondrial membrane potential) are also described, but present less relevance in the following topic and thus will not be described in detail.

## 3. Modulation of Cell Proliferation and Cell Death by *Thymus* spp. Extracts

Belonging to the Lamiaceae family, the *Thymus* genus is comprised of about 400 species, with the highest occurrence of species around the Mediterranean Sea basin, but with a worldwide distribution [68,69]. This genus’ main representative is *Thymus vulgaris* L., the most consumed and therefore the most studied thyme worldwide [70] Nevertheless, the *Thymus* genus is comprised of many other species with market value such as *Thymus mastichina* L. [71], *Thymus zygis* Loefl. ex L. [72], *Thymus* × *citriodorus* (Pers.) Schreb. [70] or *Thymus fragrantissimus* [73].

Regarding their bioactivities, the essential oils obtained from *Thymus* spp. are often used to describe the pharmaceutical potential of this genus, in detriment of its extracts (e.g., aqueous decoctions, infusions, or hydroalcoholic). In fact, thyme extracts are present in the formulations of pharmaceutical products (e.g., anti-cough and expectorant syrups [72]), and have been addressed for their anti-inflammatory [74], antimicrobial [75], anti-diabetic [76], or neuroprotective [77] activities, for example, and the most relevant in the context of this review, the cell-protection or anti-proliferative activities.

A hydroethanolic (20:80; *v*/*v*) extract of *Thymus caramanicus* Jalas, a species used in traditional medicine as an antimicrobial, anti-rheumatic and to treat skin disorders, was evaluated as a protective agent against hyperglycemia-induced apoptosis. Although using a hydroalcoholic solvent in the extraction, is known to be effective in the extraction of polyphenols [78], the authors only report the GC-MS analysis of terpenoids, with the identification of carvacrol, thymol, borneol, cymene, and γ-terpinene [79]. Using rat pheochromocytoma cells (PC-12), it was reported that *T. caramanicus* extract (60 and 80 µg/mL) reduced the toxicity induced by high glucose (100 mM). The effect was correlated with a decrease in cleaved caspase-3, cyt c release, and Bax/Bcl-2 ratio, when compared to cells exposed to 100 mM of glucose, thus confirming that *T. caramanicus* extract prevented the apoptotic cascade. The extract (100 and 150 mg/kg) was also administered to streptozotocin-induced diabetic Wistar rats, aiming to observe the reproducibility of the result in vivo. As verified in PC-12 cells, the expression of cleaved caspase-3, cyt c release, and Bax/Bcl-2 ratio were reduced in the dorsal portion of the lumbar spinal cord [79].

*Thymus serpyllum* L. methanolic extract was evaluated for its antiproliferative activity against human breast cancer cell lines MCF-7 (human breast adenocarcinoma cells) and MDA-MB-231 (human triple-negative breast adenocarcinoma cells), and MCF-10A (non-tumoral breast cells) as reference [80]. Primarily, while not presenting toxicity to MCF-10A cells, *T. serpyllum* extract exerted cytotoxicity in both MCF-7 and MDA-MB-231 cells, in concentrations ≥10 µg/mL (72 h exposure), being MDA-MB-231 more sensitive to the extract-induced cytotoxicity. In MDA-MB-231 cells, *T. serpyllum* extract ≥10 µg/mL induced DNA fragmentation and increased caspase-3 and -7 activities, corroborating with apoptosis induction and the mechanisms of action were related to a reduced expression of DNA methyltransferase and histone deacetylase, key targets in tumorigenesis regulation [80].

*T. vulgaris* anti-proliferative activity was already addressed, N. Adham, et al. [81] reported the cytotoxicity of butanol, ethyl acetate, chloroform, and hexane extract of *T. vulgaris* in two leukemia cell lines (CCRF-CEM and CEM/ADR5000; human acute lymphoblastic leukemia cells), and in various human multiple myeloma cell lines, from which NCI-H929 were highlighted as the most sensitive to *T. vulgaris* extracts. In all cell lines, chloroform extract was the most cytotoxic and thus was used to assess the mechanisms of such anti-proliferative activity [81]. Using annexin V-FITC/PI double staining assay, it was observed that *T. vulgaris* chloroform extract (5.2–25.9 µg/mL: 48 h and 72 h exposure) induced late apoptosis in NCI-H929 cells, in agreement with the microscopic observation of apoptotic bodies, DNA fragmentation, and cell shrinkage. Additionally, it was also observed depolarization of mitochondrial membrane potential and slight cell cycle arrest (G2/M phase). Cell death was related to ROS increase, however, the authors also reported increased expression of LC3-II and Beclin-1 in NCI-H929 cells, both markers of autophagy. In addition, the authors report that the cytotoxic effect of *T. vulgaris* chloroform extract was decreased when using the ferroptosis inhibitors ferrostatin-1 and deferoxamine, thus also inferring that ferroptosis is partly involved in the antiproliferative activity [81].

A human breast duct carcinoma cell line (T-47D) was also used to evaluate the anti-proliferative activity of an ethanolic extract of *T. vulgaris* [82]. As the main findings, *T. vulgaris* extract (10 and 20 µg/mL; 24 h exposure) arrested the cell cycle arrest in the S phase and induced apoptosis in T-47D cells. Cells exposed to *T. vulgaris* extract presented increased expression of proteins p53 and 14-3-3 (these proteins are key intervenients in cell cycle regulation) [82]. Additionally, the extract was not cytotoxic to Vero cells (non-tumoral monkey kidney cells), providing an insight into these extracts’ safety to normal cells [82]. Another study also analyzed the effect of a *T. vulgaris* hydroethanolic (20:80; *v*/*v*) extract in T-47D cells, showing induced apoptosis, mainly early apoptosis, in cells exposed to ~100 µg/mL for 24 h [83].

Due to the normal consumption of medicinal and aromatic plants, such as thyme, the study of its impact on the intestinal tract, using extraction methods approximate to human consumption (e.g., infusions, decoctions, condiments) is of major importance. Within this topic, Al-Menhali, et al. [84] exposed human colon carcinoma cells (HCT116) to aqueous extracts of *T. vulgaris*, verifying that at 400 and 600 µg/mL the extracts increased caspase-3 and -7 activities. In addition, reduced cell adhesion was observed at concentrations ≥200 µg/mL, and reduced cell invasion and migration were observed when HCT116 cells were exposed to 400 µg/mL of *T. vulgaris* aqueous extracts [84]. Martins-Gomes, et al. [85] reported the effect of aqueous extracts of *Thymus carnosus* Boiss. in colorectal cancer cell proliferation. Caco-2 cells exposed to *T. carnosus* aqueous extracts showed cell cycle arrest in S and G2/M phases when exposed to 400 and 500 µg/mL of *T. carnosus* aqueous decoction extract, and significant staining for late apoptosis (Annexin V-FITC/PI double staining assay). When exposing Caco-2 cells to *T. carnosus* hydroethanolic (20:80; *v*/*v*) extract [84], which contains a higher concentration of phenolic compounds and high quantities of oleanolic (OA) and ursolic acid (UA) [78,84], significantly higher toxicity was observed (IC_50_ hydroethanolic extract = 32 µg/mL vs. IC_50_ aqueous extract = 510 µg/mL; 24 h exposure) accompanied with a cell cycle arrest in G0/G1 phase (at 30–40 µg/mL). However, *T. carnosus* hydroethanolic extract induced only a slight increase in apoptotic cells, revealing that different mechanisms of cell death may be occurring as a result of different phytochemical compositions [84].

Many studies reported apoptosis induction by thyme extracts. Nevertheless, a *T. vulgaris* hydroethanolic extract (*v*/*v* % not specified), was shown to induce necrosis in human lung carcinoma cells (H460) [86], while having no effect in the cell cycle, at 3 mg/mL (24 h exposure), the extract induced DNA damage. In Annexin V-FITC/PI double staining assay, it was observed that almost the total of cells shifted from negative to both probes, to only PI-positive quadrant, meaning that the cells present no phosphatidylserine externalization (a hallmark of apoptosis), but stained with PI which is standard staining for necrotic cells [86].

As stated above, natural compounds may modulate cell death pathways, but also cell survival. *T. vulgaris* is a prime example of various protective bioactivities. A hydroethanolic extract (30:70; *v*/*v*; containing CA, RA, and luteolin-7-*O*-glucoside (L-7-G)) successfully reduced sodium nitrate induced-hepatic injury [87]. Another ethanolic *T. vulgaris* extract administered to lead-intoxicated Sprague-Dawley rats was able to reduce hepatic and renal damage [88].

Various extracts of *Thymus sipyleus* Boiss. (50–200 µg/mL), namely decoction, infusion, ethanol, or *n*-hexane, were able to reduce H_2_O_2_-induced cytotoxicity in mouse fibroblasts (3T3-Swiss albino), in both pre-incubation and co-incubation assays [89]. In addition, part of the extracts increased cell proliferation and wound closure, with the promising result when using decoction or infusion extracts, with the authors highlighting the presence of L-7-G [89].

Most studies approach thyme extracts as anti-tumoral agents when compared to studies where extracts promote non-tumoral cell proliferation. Nevertheless, even studies reporting anti-tumoral activity require further analysis of pathways involved in cell death modulation. Thus, an approach to understanding the effects of *Thymus* spp. extracts and predict the extract’s potential as an anti-tumoral agent may arise from its phytochemical composition. The phytochemical profiles of species from the *Thymus* genus can present great variation, as genetic variations between species, the vegetative stage, and edaphoclimatic factors play a significant role in their composition [90,91,92,93]. Even within a single species, the concentration of an individual component can vary greatly, as reported for RA in *Thymus longicaulis* C. Presl. hydromethanolic extracts (50:50; *v*/*v*), which ranged between 12.97–3029.56 µg/mL when compared to harvests from different seasons [94]. Nevertheless, some phytochemicals tend to be consistently identified in *Thymus* spp. extracts, as seen in Table 1.

**Figure 4:**
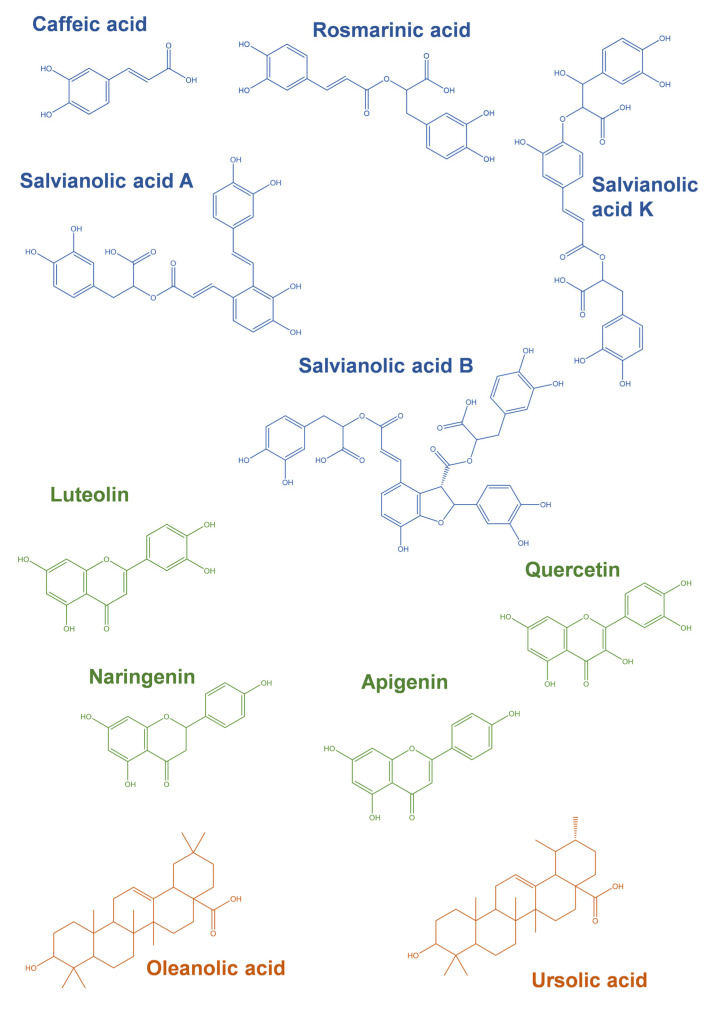
Chemical structures of common phytochemicals identified in *Thymus* spp. extracts. Blue: phenolic acids; green: flavonoids; orange: pentacyclic triterpenoids.

The most common phytochemicals described in aqueous or hydroalcoholic extracts of *Thymus* spp. usually fall under phenolics and terpenoids classes, and some examples of compounds and their structures are illustrated in Figure 4. The first class is usually represented by phenolic acids and flavonoids.

As seen in Table 1, from the species listed, all species present RA (Figure 4) in its composition, an ester of CA, and salvianic acid A [105], being the most common polyphenolic in this genus’ extracts. *Thymus algeriensis* L. even presents a glycoside derivative of RA (Table 1). Among phenolic acids, CA (Figure 4) presents an almost ubiquitous dispersion through *Thymus* spp. extracts, but it is not a major compound, in contrast with salvianolic acids (SAs). Considering the most common phenolics of this group, salvianolic acid A (SAA), I (SAI), and K (SAK), SAs can be found in most thyme species, as well as many of its isomers.

Although, as observed in Table 1, extracts from various species have not been described for their SAs content (e.g., *Thymus sibthorpii* Benth. [95] or *Thymus praecox* Opiz [95]), it can’t be excluded the existence of these phenolic acids in these species, as the use of different extraction methods, and the influence of edaphoclimatic factors may unveil new phytochemical profiles in future publications.

In the present review, we also highlight the relevance of glycoside derivatives of common flavonoids found in extracts from *Thymus* spp., namely apigenin, chrysoeriol, eriodictyol, luteolin, naringenin, and quercetin. Luteolin derivatives are present throughout all the extracts listed in Table 1. Luteolin derivatives present a great range of sugar residues, mainly *O*-linked, and with -hexoside and -hexuronide as the most common substitutions, but -hexoside-pentoside, -pentoside, -acetyl-pentosyl-hexoside, -acetyl-hexoside, -dihexoside, -dipentoside or -rutinoside were also identified [67,76,97]. Most authors perform LC-MS analysis of the extracts (e.g., [60,66,67]), allowing the identification of the derivative, but not the *O*-link position or the exact sugar residue. In fact, -hexoside and -hexuronide residues are the most common for all the flavonoid derivatives listed in Table 1. Nevertheless, apigenin also presents a very common derivative, vicenin-2, in various thyme extracts, for example, in *T. carnosus* [78], *T. mastichina* [71], *T. vulgaris* [70], or *T. zygis* [70]. Vicenin-2 is apigenin-(6,8)-*C*-diglucoside, thus being the major difference in the *C*-link between the aglycone and the sugar residues. Eriodictyol and quercetin (Figure 4) also contribute greatly to the presence of glycoside derivatives in thyme extracts. Among them, the specific derivates quercetin-3-*O*-glucoside, querctin-3-*O*-glucoronide, and querctin-3-*O*-rutinoside have been identified in *Thymus* spp. extracts [101,106]. Naringenin and chrysoeriol -hexoside and -hexuronide are also common within the *Thymus* genus, but nevertheless often present at residual levels.

Apart from phenolic components, a second group of compounds is often identified in *Thymus* spp. extracts, the pentacyclic triterpenoids. Unable to be extracted with water and thus not present in aqueous extracts [78], both OA and UA have been identified in various thyme extracts, as observed in Table 1. In fact, other terpenoids have been identified in methanolic extracts of *T. sibthorpii, T. serpyllum*, *T. praecox*, *Thymus pulegioides* L., *T. longicaulis* and *Thymus austriacus* Bernh. ex Rchb. in various vegetative phases [95], in addition to OA and UA (Figure 4), corosolic and betulinic acids have also been identified and quantified in these species. For example, in *T.* × *citriodorus* only OA and UA have been identified [95], but as discussed above, future studies regarding the conditions of plants’ growth or harvest may update the knowledge regarding the phytochemical profiles known until now.

The phytochemical composition is deeply connected to the bioactivities displayed by the extracts described above in this section. Therefore, in the next section, it will be reviewed and discussed the effect of individual phenolic acids, *O*-link derivatives of flavonoids, and pentacyclic triterpenoids commonly found in *Thymus* spp. extracts with respect to their effect on modulating cell death and survival pathways.

## 4. Modulation of Cell Survival and Cell Death Pathways by Phytochemicals Present in *Thymus* spp. Extracts

Similar to the effects of the whole extract of *Thymus* spp., some of its major constituents have also been studied for their ability to modulate both cell survival and cell death. The latter is evaluated mainly to ascertain its potential anti-tumor activity, analyzing mostly cell death induction in in vitro cancer models. Cell survival is often reported as the ability to protect normal cells against negative stimuli induced by xenobiotics, which may arise from pathogens or toxins, for example. The following sections described reports of these modulations induced by compounds that are described as the main phytochemicals present in extracts of *Thymus* spp. For all studies, it is reported the concentration(s) used in each assay, the experimental model (mostly cell line models and in vivo studies using rat/mouse), and the main observations regarding cell survival vs. death modulation.

### 4.1. Cellular Protection

Medicinal and aromatic plants, such as some of the species of thyme described above (e.g., *T. vulgaris*, *T. mastichina*, *T. × citriodorus*), and their phytochemicals, are consumed through the diet regularly. Ideally, it is intended that they do not disturb the proliferation/death pathways in a healthy situation. For this reason, most studies address the ability of the phytochemicals to stimulate cell survival after exposure to pathogens’ metabolites, toxins, and other xenobiotics. In addition, it may be desirable that cell proliferation is induced after injuries caused by trauma, often addressed as wound-healing activity.

Regarding caffeic acid’s protective effects, it is worth highlighting the different mechanisms in which it interferes (Table 2). Whilst in human neuroblastoma cells (SH-SY5Y) [107] exposed to cyclophosphamide it was observed that CA (400 µM) clearly inhibited the intrinsic pathway of apoptosis, confirmed by increased expression of anti-apoptotic protein Bcl-2, and reduced expression of Bax, caspase-3 as well as decreased cyt c release [107], in Human peripheral blood mononuclear cells [108], CA (60–120 µM) inhibition of H_2_O_2_-induced apoptosis was Bcl-2 independent, thus revealing different effects depending on the apoptosis-inducing agent or the cell line model used. Even more, in human monocytic cells (U937), although being lymphoma cells, CA (50 µM) inhibited the NF-kB pathway and tyrosine kinase activity in ceramide-induced apoptosis, thus inferring a different mechanism of action [109].

As observed for CA, anti-apoptotic reports were also published in experimental models using RA (Table 2). Overall, studies using RA provide more data regarding the signaling pathways involved, when compared to CA. At 56 µM, RA reduced H_2_O_2_-induced apoptosis in SH-SY5Y, confirmed through the downregulation of the pro-apoptotic proteins Bax and caspase-3, and the upregulation of Bcl-2. RA also induced the expression of heme-oxigenase-1 (HO-1), a protein relevant for an intracellular antioxidant response, via PKA (protein kinase A) and PI3K pathways [110].

In mouse proximal tubular epithelial cells exposed to cadmium and RA (40 µM), the polyphenol reduced cyt c release, and the cleavage of caspase-3, -8, and -9, lowered the expression of APAF-1, NF-kB, PKC, and TNFR2, as well as reduced FAS (apoptosis antigen 1) activation [111]. All these biomarkers supported the RA effect in preventing cadmium-induced apoptosis [111]. Using rat fibroblasts (RL-3A), it was also reported that low concentrations of RA (6.25–50 μM) reduced acrylamide-induced apoptosis, with a reduction of Bax/Bcl-2 ratio and reduction of caspase-3 activation [112].

Among salvianolic acids, SAA, SAB, and SAC are the most addressed concerning bioactivity studies. As observed for CA and RA, also SAA and SAB were able to reduce H_2_O_2_-induced apoptosis. SAA (50 μM) upregulated mTORC1 (mammalian target of rapamycin complex 1) pathway and downregulated MAPK pathway to protect human retinal pigmented epithelium cells (ARPE-19) from H_2_O_2_-induced apoptosis. The antioxidant mechanism was mediated by Nrf2 (nuclear factor erythroid 2-related factor 2) and HO-1 activation, and thus SAA may be targeting the Akt/mTORC1/Nrf2/HO-1 signaling axis [113]. Regarding SAB, the cell-protective effect was observed at 20 μM, a concentration lower than the ones reported from CA (60 and 120 μM), RA (56 μM), or SAA (50 μM) (Table 2), although being used different cell lines. SAB protected rat cerebral microvascular endothelial cells against H_2_O_2_-induced apoptosis through PI3K/Akt/Raf/MEK/ERK axis modulation [114].

Considering cell death pathways, apart from apoptosis, it is also relevant to mention an in vivo study using SAB (25–50 mg/kg) administered to Sprague Dawley rats, in which SAB was able to prevent ferroptosis in a model of myocardial infarction, confirmed through increased expression of ferroptosis biomarkers cystine/glutamate transporter (xCT), glutathione peroxidase 4, ferroportin 1 (FPN1) and ferritin heavy chain (FTH1) when compared to animals with myocardial infarction [115].

Salvianolic acid C (SAC) was also described for its potential modulation in cell survival. Studied both in vivo, using ICR (Institute of cancer research [116]) mice administered with 20 mg/kg of SAC followed by liver tissue analysis, and in vitro, using human periodontal ligament stem cells (exposed to 5 mM SAC [117]), SAC was able to reduce Bax and caspase-3 expression in acetaminophen-induced toxicity [116] and in LPS-induced apoptosis [117].

In addition to phenolic acids, flavonoids also represent a significant portion of the polyphenols present in *Thymus* spp. extracts, and, as seen in Table 1, are frequently present as a wide range of glycoside derivatives. Nevertheless, most studies address the effect of aglycones and not their derivatives. In the case of luteolin, for example, its wound-healing activity is already described in normal mouse fibroblasts (3T3 cells), with increased proliferation and migration [118], but also in vivo, where it enhanced the healing and skin’s re-epithelization of the wound in streptozotocin (STZ)-induced diabetic rats, presenting the potential for the treatment of skin wounds caused by diabetes [119]. Similarly to phenolic acids, described in Table 2, luteolin is also able to prevent H_2_O_2_-induced apoptosis [120]. H9C2 cells (rat myoblasts) pre-exposed to 10 μM of luteolin presented significantly lower H_2_O_2_-induced apoptosis, with an increase in Bcl-2 and Akt expression, and reduced expression of Bax, caspase-3, caspase-8, and p53, when compared to H_2_O_2_ control [120]. Among luteolin derivatives, luteolin-(?)-*O*-hexoside is one of the most common among thyme extracts (Table 1), and the specific derivative L-7-G has been identified in various *Thymus* spp. [87,89]. This derivative is also the most studied among luteolin derivatives (Table 2 and Table 3). In H9c2 cells, luteolin played a protective role [120], as 10–20 μM luteolin reduced hypoxia/reoxygenation-induced apoptosis through the inhibition of apoptosis (downregulation of caspase-3 and PARP) and through modulation of Fas/FasL, ERK and JNK pathways [121]. L-7-G also exerted neuroprotective activity, reducing 6-hydroxydopamine-induced apoptosis and DNA damage [122].

**Table 2:** Cell survival modulation by common phytochemicals from *Thymus* spp. extracts in in vitro and in vivo animal experimental models.

Phenolic Acid	Concentration	Experimental Model	Observations	Ref.
Caffeic acid	60 and 120 µM	Human peripheral blood mononuclear cells (PBMCs)	Reduced phosphatidylserine externalization in H_2_O_2_-induced apoptosis Reduced DNA fragmentation Reduced lipid peroxidation Bcl-2-independent mechanism	[108]
	50 µM	Human monocytic lymphoma cells (U937)	Inhibition of ceramide-induced apoptosis and NF-κB DNA-binding Tyrosine kinase inhibition	[109]
	400 µM	Human neuroblastoma cells (SH-SY5Y)	Reduced cyclophosphamide-induced apoptosis and reduced DNA damage Increased Bcl-2 and reduced cyt C, Bax, and caspase-3 expression in cells exposed to cyclophosphamide	[107]
Rosmarinic acid	56 µM	Human neuroblastoma cells (SH-SY5Y)	Reduced H_2_O_2_-induced apoptosis Downregulation of caspase-3 and Bax Upregulation of Bcl-2 and HO-1 PKA and PI3K-dependent induction of HO-1	[110]
	40 µM	Mouse proximal tubular epithelial cells	Reduced cadmium-induced apoptosis and DNA damage Reduced cyt c release, FAS activation, and the cleavage of caspase-3, -8 and -9 Reduced the expression of APAF-1, NF-kB, PKC and TNFR2	[111]
	6.25–50 μM	Rat liver fibroblasts (BRL-3A)	Reduced acrylamide-induced apoptosis Reduced ROS content Reduced Bax/Bcl-2 and cleaved caspase-3/caspase-3 ratio	[112]
Salvianolic acid A	50 μM	Human retinal pigmented epithelium cells (ARPE-19)	Reduced H_2_O_2_-induced apoptosis Reduced caspase-3 cleavage Upregulation of the mTORC1 (mammalian target of rapamycin complex 1) pathway Downregulation of MAPK pathway	[113]
	100 µg/kg	ICR mice	Protects the blood-brain barrier from apoptosis in induced ischemic brain Reduction of NF-κB pathway and cleaved caspase-3 expression Increased Bcl-2 expression	[123]
	50 µM	Rat myocardium cells (H9c2)	Reduced H_2_O_2_-induced apoptosis Restored p-JNK/JNK ratio and increased thioredoxin expression	[124]
Salvianolic acid B	25–50 mg/kg	Sprague Dawley rats	Prevented myocardial infarction-induced ferroptosis Increased the expression of cystine/glutamate transporter (xCT), glutathione peroxidase 4, ferroportin 1(FPN1), and ferritin heavy chain (FTH1)	[115]
	10 µM	Rat myocardium cells (H9c2)	Reduced hypoxia-induced apoptosis	[125]
	20 µM	Rat cerebral microvascular endothelial cells	Reduced H_2_O_2_-induced apoptosis Reduced caspase-3 and 9 activities PI3K/Akt/Raf/MEK/ERK pathway-dependent response	[114]
	10 µM	Human neuroblastoma cells (SH-SY5Y)	Reduced 6-hydroxydopamine-induced apoptosis Prevented alterations in nucleus morphology Normalized intracellular calcium concentration and PKC phosphorylation	[126]
Salvianolic acid C	20 mg/kg	Liver tissue of ICR mice	Protection against acetaminophen-induced toxicity Decreased expression of Bax, cleaved caspase-3, and cyt C release	[116]
	5 mM	Human periodontal ligament stem cells	Decreased LPS-Induced apoptosis Cell cycle modulation Increased Bcl-2 expression Decreased Bax and caspase-3 expression	[117]
Luteolin-7-*O*-glucoside	10 and 20 µM	Rat myocardium cells (H9c2)	Reduced hypoxia/reoxygenation-induced apoptosis Downregulation of caspase-3, PARP, Fas, Fasl, p-ERK1/2 and p-JNK	[121]
	1 µM	Human neuroblastoma cells (SH-SY5Y)	Reduced 6-hydroxydopamine-induced apoptosis Decreased DNA damage and caspase-3 activity	[122]
Quercetin-3-*O*-glucoside	2.15–21.5 µM	Human skin fibroblasts (TIG-108)	Prevented advanced glycation end-products-induced apoptosis	[127]
Eriodictyol-7-*O*-glucoside	30 mg/kg	Wistar rat model of cerebral ischemic injury	Reduced ischemia/reperfusion-induced apoptosis Upregulation of Nrf2, HO-1	[128]

Just as described for luteolin, quercetin also exerted wound healing activity at concentrations as low as 10 µM [129], protected against radiation-induced apoptosis [130], and against oxidative stress induced-apoptosis [120,131].

**Figure 5:**
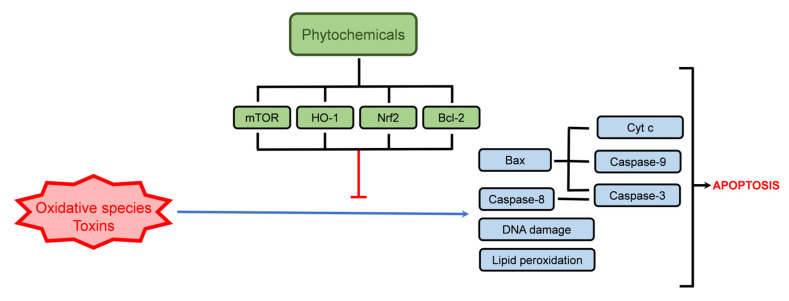
Schematization of main cellular targets of phytochemicals that participate in cell protection against xenobiotic-induced apoptosis.

Quercetin derivatives also exert anti-apoptotic activities, as observed for quercetin-3-*O*-glucoside (Q-3-G) (Table 2). Q-3-G can prevent the cytotoxicity induced by advanced glycation end-products (AGEs), in human skin fibroblasts (TIG-108), at low concentrations (2.15 µM), where the inhibition of caspase-3 and -7 activity (44% inhibition) supported Q-3-G anti-apoptotic activity [127]. In addition, another quercetin derivative, quercetin 3-*O*-malonylglucoside, also induced caspase inhibition, although being less effective (21.8% inhibition) [127]. Eriodictyol-7-*O*-glucoside (E-7-G) assumes a protective role, mainly through its capacity to activate the Nrf2 pathway, demonstrated in both an in vivo model of cerebral ischemic injury and in a cisplatin-exposed human renal mesangial cell line (HRMC), where in E-7-G reduced apoptosis induced by the negative stimuli [128,132]. Among other glycoside derivatives described in Table 1 but less abundant in thyme extracts such as naringenin derivatives, naringenin-7-*O*-glucoside, protected H9c2 cells against doxorubicin-induced apoptosis, through Bcl-2 upregulation and caspases downregulation, being a promising option to treat cardiomyopathy associated with chemotherapy drugs [133]. Figure 5 schematically shows the main cellular targets regarding the phytochemicals’ protective activity towards xenobiotic-induced apoptosis.

Nevertheless, the anti-tumoral activity is the one better described and more extensively studied when considering phytochemicals. As it will be described below, the pentacyclic triterpenoids have mainly been studied for their anti-tumor properties. These phytochemicals present anti-proliferative activity towards various cancer types, as will be discussed below.

### 4.2. Cell Death Induction

The most studied cell death mechanism is apoptosis. Given the higher availability of markers for molecular targets within the signaling cascade, the apoptotic process is the most addressed also in studies using natural products. However, the other cell death processes are being unveiled, thus providing new biomarkers to undergo studying these pathways. Among the major phytochemicals identified in *Thymus* spp. extracts, phenolic acids, glycosidic derivatives of flavonoids, and pentacyclic triterpenoids have been shown to exert anti-tumoral activity through various mechanisms. After apoptosis is the most discussed, several reports of autophagy have also been published. Despite the high variability of chemical structures presented by these classes of phytochemicals, it also can be seen in Table 3, Table 4 and Table 5 that several pathways are targets of phytochemicals, such as the Akt pathway, in which the proteins involved in the intrinsic apoptosis pathway and cell cycle regulation are frequently key targets.

#### 4.2.1. Phenolic Acids

A summary of various reports regarding the modulation of cell death pathways by CA, RA, and SAs is presented in Table 3. As seen in Section 4.1, CA was observed to protect the cell against apoptosis-inducing agents, but it also shows the capacity to induce cell death in various tumoral cells.

CA-induced apoptosis in all studies is presented in Table 3, with various markers being assessed. Regarding genotoxicity, CA (360 µM) reduced DNA biosynthesis in human tongue squamous cell carcinoma cells (CAL-27), induced DNA fragmentation in U937 cells (≥200 µM) [109], and cell cycle arrest in human melanoma cells (SK-Mel-28) at 200 µM [134], human breast adenocarcinoma cells (MDA-MB-231) at 100 µM [135], and in human squamous carcinoma cells (Detroit 562) at 100 µM [136]. Cell cycle arrest in MDA-MB-231 cells was in the S phase, while the other cell lines presented arrest in G0/G1 phase. Overall, most studies confirmed apoptosis induction through the upregulation of caspase-1, -3, -7, -8, and -9 (Table 3). In HeLa cells (human cervical cancer cells), CA induced cyt c release when cells were exposed to 10 mM of the phenolic acid, a concentration well above the remaining studies here reviewed [137].

Standing out, Chang, et al. [138] verified that CA (400–800 μM) induced Ca^2+^ release from the endoplasmic reticulum in human gastric carcinoma cells (SC-M1), but it was not connected to apoptosis, despite the potential role of endoplasmic reticulum in apoptosis [138]. In human tongue squamous cell carcinoma cells (CAL-27) exposed to 360 µM of CA, apoptotic events were related to proline dehydrogenase (PODH) activity [139]. This enzyme catalyzes mitochondrial degradation of proline and is linked to homeostasis and carcinogenesis inhibition. Frequently downregulated in tumoral cells, PODH can induce both intrinsic and extrinsic apoptosis. At 360 µM CA induced an increase in PODH expression, accompanied by an increase in p53 expression, which promotes PODH expression [139].

Concerning RA’s pro-apoptotic activity, a significant number of studies addressed the signaling pathways triggered or inhibited, in various experimental models. As observed for CA, U937 cells exposed to RA (60 μM) also undergo apoptosis, with an increase in cleaved PARP and cyt c release indicating the activation of the intrinsic pathway [140]. RA exhibits good potential as an anti-proliferative agent against breast cancer.

**Table 3:** Cell death modulation by common phenolic acids from *Thymus* spp. extracts in in vitro and in vivo animal experimental models.

Phenolic Acid	Concentration	Experimental Model	Observations	Ref.
Caffeic acid	200 µM	Human melanoma cells (SK-Mel-28)	Induced apoptosis Cell cycle arrest (G0/G1 phase) Increased caspase-1, -3, and -8 genes expression	[134]
	100 µM	Human breast adenocarcinoma cells (MDA-MB-231)	Induced apoptosis Cell cycle arrest (S phase)	[135]
	10 mM	Human cervical cancer cells (HeLa)	Induced apoptosis Increased cleaved caspase-3 expression Induced cyt c release Downregulation of Bcl-2 Upregulation of p53	[137]
	≥200 µM	Human monocytic lymphoma cells (U937)	Apoptosis induction DNA fragmentation	[109]
	300 µM	Human cervical adenocarcinoma cells (HeLa)	Anti-proliferative activity mediated by caspase-3, -7, -9 pathways	[141]
	300 µM	Human cervical carcinoma cells (CaSki)		
	100 µM	Human squamous carcinoma cells (Detroit 562)	Induced apoptosis Cell cycle arrest (G0/G1 phase)	[136]
	400–800 μM	Human gastric carcinoma cells (SC-M1)	Apoptosis induction Ca^2+^-independent pathway	[138]
	360 µM	Human tongue squamous cell carcinoma cells (CAL-27)	Proline dehydrogenase-dependent apoptosis Decreased DNA biosynthesis p53 and cleaved caspase-9 upregulation	[139]
Rosmarinic acid	60 μM	Human monocytic lymphoma cells (U937)	Increased TNF-α-induced apoptosis and DNA fragmentation Increased caspase-3 and -8 activity, PARP cleavage, and cyt c release Inhibited NF-kB pathway with TNF-α exposure	[140]
	60 μM	Human breast adenocarcinoma cells (MCF-7)	Increased TNF-α-induced apoptosis	[140]
	60 μM	Human hepatic carcinoma cells (HepG2)	Increased TNF-α-induced apoptosis	[140]
	200 and 400 μM	Human glioma cells (U251 and U343)	Induced apoptosis Reduction of PI3K, p-Akt, NF-κB, Fyn (Proto-oncogene tyrosine-protein kinase Fyn), and Bcl-2 expression Increased Bax and cleaved caspase-3 expression Reduced cell migration	[142]
	200 μM	Human prostate adenocarcinoma cells (PC-3)	Induced apoptosis and DNA fragmentation Upregulation of p53, p21, caspase-3, cleaved PARP-1 and Bax Downregulation of histone deacetylase 2 (HDAC2), Bcl-2, cyclin D1 and cyclin E1	[143]
	200 μM	Human prostate carcinoma cells (DU145)	Induced apoptosis and necrosis Induced DNA fragmentation Upregulation of p53, caspase-3 and Bax Downregulation of histone deacetylase 2 (HDAC2), Bcl-2, cyclin D1 and cyclin E1	[143]
	125–400 μM	Human triple-negative breast adenocarcinoma cells (MDA-MB-231)	Induced apoptosis Cell cycle arrest (G0/G1 phase) Upregulation of *HRK* (activator of apoptosis harakiri), *TNFRSF25* (TNF receptor superfamily member 25), and *BNIP3* (Bcl-2 interacting protein 3) genes Downregulation of *TNFRSF11B* (TNF receptor superfamily member 11b) gene	[144]
	125–400 μM	Human triple-negative breast adenocarcinoma cells (MDA-MB-468)	Induced apoptosis Cell cycle arrest (S phase) Upregulation of *TNF*, *GADD45A* (Growth Arrest and DNA damage-inducible alpha), and *BNIP3* genes Downregulation of *TNFSF10* (TNF Superfamily Member 10) and *BIRC5* (Survivin/Baculoviral inhibitor of apoptosis repeat containing 5) genes	[144]
	100 µM	Human colorectal adenocarcinoma cells (HCT-15)	Induced apoptosis Downregulation of phospho-ERK pathway	[145]
	100 µM	Human colorectal adenocarcinoma cells (CO115)	Induced apoptosis	[145]
Salvianolic acid A	50 µM	Human acute monocytic leukemia cells (THP-1)	Induced apoptosis Increased cleaved caspase-3 and cleaved PARP Decreased Bcl-xL and p-Akt expression	[146]
	50 µM	Human acute myelogenous leukemia cells (KG-1)		
	50 µM	Human acute myeloblastic leukemia cells (Kasumi-1)		
Salvianolic acid B	100 and 200 µM	Human hepatic adenocarcinoma or endothelial cells (SK-Hep-1) *	Induced apoptosis and autophagy Loss of mitochondria membrane depolarization Increased expression of cleaved caspase-3, cleaved caspase-9, and cleaved PARP Increased cyt C release Decreased p-Akt expression	[147]
	100 and 200 µM	Human liver carcinoma or HeLa derivative (Bel-7404) *		[147]
	200 µM	Human colorectal carcinoma cells (HCT116)	Induced apoptosis and autophagy Formation of autophagosomes and expression of LC3 Increased expression of cleaved caspase-3 and -9, and cleaved PARP Decreased expression of p-Akt and p-mTOR	[148]
	200 µM	Human colorectal adenocarcinoma cells (HT29)		
	10–100 µM	Human glioma cells (U87)	Induced apoptosis Increased expression of cleaved caspase-3, p-p38 and p-p53	[149]

Notes: “p-“ phosphorylated; *-cell lines with classification issues.

In MCF-7 cells, RA (60 μM) potentiated TNF-α-induced apoptosis [140]. In triple-negative breast cancer cells (MDA-MB-231 and MDA-MB-468), RA (125–400 μM) induced cell cycle arrest and apoptosis, but in a cell-dependent pattern [144]. In MDA-MB-231 cells, the cell cycle arrest was observed in G0/G1 phase, while MDA-MB-468 cells were shown to accumulate in the S phase [144]. In addition, in MDA-MB-231 cells the genes *HRK* (activator of apoptosis harakiri), *TNFRSF25* (TNF receptor superfamily member 25) were upregulated, in contrast with the genes *TNF* and *GADD45A* (growth arrest and DNA damage-inducible alpha), which were upregulated in MDA-MB-468 cells. The gene *BNIP3* (Bcl-2 interacting protein 3) was upregulated in both cell lines [144].

In human prostate cancer cell lines PC-3 (adenocarcinoma) and DU145 (carcinoma), RA (200 μM) induced apoptosis and DNA damage [143]. Regarding cell cycle control, in both cell lines, decreased expression of histone deacetylase 2 (HDAC2), cyclin D1, and cyclin E1 was observed. Concerning cell death, the intrinsic pathway is involved in RA’s anti-proliferative activity, as a decrease in Bcl-2 levels was observed in both cell lines, as well as an increase in Bax and caspase-3 [143]. Noteworthy, p53 was upregulated in both PC-3 and DU145 cells exposed to RA, being this protein most likely to be the inducer of Bax increase and of cyclins downregulation [143].

Concerning tumoral cell migration and invasiveness, RA (200–400 μM) reduced human glioma cells (U251 and U343) migration and invasion [142]. Similar findings were observed in human melanoma cells (A375), with reduced migration and invasion, where RA (555 μM) inhibited ADAM17 (disintegrin and metalloprotease 17), EGFR (epidermal growth factor receptor), p-Akt and p-GSK3β (glycogen synthase kinase-3 β) expressions [150].

As described above in Section 4.1, the Akt pathway is a key target for the action of various phytochemicals, for example, for SAB that protects against H_2_O_2_-induced apoptosis. In fact, the Akt pathway is highlighted in various studies regarding SAs, both toward cell survival and cell death. In human acute myeloblastic leukemia cells (THP-1, KG-1, and Kasumi-1), a decrease in Akt phosphorylation was observed in SAA-induced apoptosis (50 μM), confirmed through the decrease in Bcl-xL expression and an increase in cleavage caspase-3 and PARP [146]. In human colorectal cancer cell line models, HCT116 and HT29, SAB (200 μM) also decreased Akt phosphorylation and increased caspases (-3/-9) and PARP cleavage, resulting in apoptosis [148]. In addition, the occurrence of autophagy, by elevated levels of LC3 was also observed [148]. In hepatic carcinoma cells SK-Hep-1 and Bel-7404 (both cell lines with classification issues, also described endothelial cells and HeLa-cell contaminated/HeLa-derivative, respectively [151]), SAB (200 μM) also induced apoptosis and autophagy [147]. Mitochondria-dependent apoptosis was observed through the increases in cleaved caspase-3, cleaved caspase-9, cleaved PARP, and cyt c release [147]. Increased autophagosome count, as well as increased LC3 and Beclin-1 expressions, confirmed autophagy [147], thus revealing various simultaneous cell death pathways. As described above, the Akt pathway was also significant in the signaling cascade triggered by SAB, as p-Akt was significantly decreased [147].

As observed in Table 2, SAs also present the potential to modulate cell survival, in addition to their anti-tumoral activity. These studies highlight the potential role of SAs in survival vs. death pathways modulation. As seen in Table 1, *Thymus* spp. is a source of various SAs and several isomers of each of them, thus being an understudied subclass of phenolic compounds.

#### 4.2.2. Glycoside Derivatives of Flavonoids

Flavonoid glycoside derivatives have also been addressed for their anti-tumoral activity, as summarized in Table 4. Nevertheless, as described above most studies are towards the aglycones, highlighting the lack of studies concerning these derivatives.

Luteolin was shown to exert anti-tumoral activity, described in various cell lines, such as in colorectal cancer cells (HT-29), where it induced depolarization of mitochondrial membrane potential, increased cyt c release, Bax expression, caspase-3, and caspase-9 activation, and reduced Bcl-2 expression, while presented no toxicity to a normal colorectal cell line (FHC) [152]. The pro-apoptotic activity was also observed in melanoma [153], glioblastoma [154], and breast cancer [155] cells.

Another similarity to the phenolic acids is luteolin’s ability to induce autophagy and modulate the Akt pathway. In SMMC-7721 cells (cells used as hepatocellular carcinoma but identified as a potential HeLa derivative [151]), 100 μM of luteolin-induced cell cycle arrest in G0/G1, and apoptosis at concentrations ≥25 μM, accompanied by increased Bax/Bcl-2 ratio [156]. Autophagy involvement in cell death was confirmed through increased expression of LC3-II and beclin-1, as well as by an increase in autophagosome count [156]. In contrast, luteolin also inhibited autophagy in a model of ovalbumin-induced asthma using Balb/c mice [157]. In lung tissue, luteolin decreased LC3 and beclin-1 expression, while increasing p62 expression, in an effect caused by PI3K/Akt/mTOR axis activation [157].

**Table 4:** Cell death pathways modulation by common flavonoid derivatives found in *Thymus* spp. extracts using in vitro and in vivo animal experimental models.

Flavonoid	Concentration	Experimental Model	Observations	Ref.
Luteolin-7-*O*-glucoside	80 µM	Human nasopharyngeal carcinoma cells (NPC-039 and NPC-BM)	Induced apoptosis Cell cycle arrest (S and G2/M phases) Increased DNA condensation Increased FAS, TNFR1, RIP (ribosome-inactivating protein), DR5 (death receptor 5), cleaved caspase-3, 8 and -9, Bax, t-BID, cleaved PARP and p21 expression Reduced Bcl-xL and Bcl-2 expression Modulation of Akt pathway	[158]
	200 µM	Human hepatic carcinoma cells (HepG2)	Induced apoptosis and DNA damage Condensed chromatin and apoptotic bodies Increased cleaved PARP expression Upregulation of the JNK pathway Caspase-independent mechanism Cell cycle arrest (G2/M phase)	[159]
	120 µM	Human colorectal adenocarcinoma cells (COLO 320 DM)	Induced apoptosis Reduction of β-catenin expression	[160]
Quercetin-3-*O*-glucoside	100 µM	Human hepatic carcinoma cells (HepG2)	Induced apoptosis Cell cycle arrest (S phase) Inhibition of DNA topoisomerase II Increase caspase-3 activity	[161]
Quercetin-3-*O*-glucuronide	40 and 60 µM	Human embryonic neural stem cells	Increased cell proliferation and migration Increased p-Akt/Akt ratio Increased cyclin D1 and Brain-derived neurotrophic factor (BDNF) expression Increased C-X-C chemokine receptor type 4 gene (*CXCR4*) expression	[162]
	50 µM	Human hepatic carcinoma cells (HepG2)	Reduced doxorubicin resistance Increased DOX-induced apoptosis	[163]
	100 µM	Human breast adenocarcinoma cells (MCF-7)	Induced apoptosis Cell cycle arrest (S phase)	[164]
Quercetin-3-*O*-glucuronide + Quercetin-7-*O*-glucuronide + Quercetin-4′-*O*-glucuronide	2.5–10 µM	Human lung carcinoma cells (NCL-H209)	Induced apoptosis Cell cycle arrest (S and G2/M phases) Increased caspase-3 activity Increased p21, Bak, and Bax expression Increased cyt C release Reduced Bcl-2 expression	[165]
Apigenin-7-*O*-glucoside	25–100 µM	Human gastric adenocarcinoma cells (AGS)	Induced pathway apoptosis Increased expression of cleaved caspase-3, -8 and PARP, FasL Fas Induced autophagy Increased expression of LC3, p-JNK, Beclin-1 and p62 Cell cycle arrest (G2/M phase) Reduced expression of cyclin B1, M-phase inducer phosphatase 3 (CDC25C), Cyclin-dependent kinase 1 (CDK1), p-PI3K, p-Akt and p-mTOR	[166]

Among luteolin glycoside derivatives, L-7-G’s potential to induce apoptosis was demonstrated in nasopharyngeal [158], hepatic [159] and colorectal [160] carcinoma cell line models. In both nasopharyngeal carcinoma [158] and hepatic carcinoma [159] exposure to L-7-G cell cycle arrest in the G2/M phase was observed. Results in human nasopharyngeal carcinoma cells (NPC-039 and NPC-BM) provide a significant contribution to the pathways involved when cells are exposed to 80 µM of L-7-G, reporting the reduced expression of anti-apoptotic proteins Bcl-2 and Bcl-xL, and the higher expression of FAS, TNFR1, RIP, DR5, cleaved caspase-3, 8 and -9, Bax, t-BID and cleaved PARP, indicating the involvement of mitochondria in the apoptosis [158]. In addition, the Akt pathway also played a significant role, as observed above for other polyphenols [158]. Interestingly, although exposure of HepG2 cells to 200 µM L-7-G induced PARP cleavage, apoptotic bodies, and DNA damage, the authors reported that the cell death was caspase-independent, as no alterations were observed in caspase-3, -8, and -9 expression [159].

Quercetin pro-apoptotic activity is also reported, as the flavonoid-induced apoptosis in lung carcinoma cells (A549) [167], gastric carcinoma cells (BGC-823) [168], breast adenocarcinoma cells (MCF-7) [169], acute promyelocytic leukemia cells (HL-60) [170] and cervical cancer cells (HeLa) [171]. In addition to apoptosis, quercetin was also able to induce autophagy in gastric cancer cells [172] and breast cancer cells [173]. In fact, breast cancer cells’ sensitivity to quercetin was well explored, as the flavonoid modulates various pathways, being able to reduce cell mobility [173] for example, and in addition, the induction of necroptosis by quercetin may play a role in the cytotoxicity observed [174].

Q-3-G anti-tumoral activity was reported in HepG2 cells, where 100 µM increased caspase-3 activity in a time-dependent manner (increased in 48 h exposure vs. 24 h exposure) and induced cell cycle arrest in the S phase. The mechanism of action was related to topoisomerase II catalytic inhibition, thus modulating DNA replication and condensation [127]. Quercetin-(?)-*O*-glucuronides were also studied for their anti-tumoral activity, mainly quercetin-3-*O*-glucuronide (Q-3-GR), which presents potential as an adjuvant of chemotherapy drugs. Q-3-GR is capable of inducing apoptosis and cell cycle arrest in breast cancer cells (MCF-7) at 100 µM [164], and is also able to potentiate the doxorubicin (DOX) anti-tumoral effect by reducing HepG2 cell drug resistance [163]. Complementing this effect, Q-3-GR was shown to partially protect human kidney tubular cells HK-2 and rat kidney cells (NRK-52E) from cisplatin toxicity, thus supporting that Q-3-GR can act as apoptosis-inducer in tumoral cells, enhancer of chemotherapy drugs, and provides protection to non-target tissues. Akt pathway modulation plays a significant role in Q-3-GR bioactivities. While the potentiating effect of Q-3-GR (50 and 100 µM) in doxorubicin was mediated, in part, by inhibition of Akt and PI3K phosphorylation [163], thus favoring apoptosis pathways, in human embryonic neural stem cells, 40 and 60 µM Q-3-GR were shown to increase Akt phosphorylation, which contributed to increased cell proliferation [162]. These studies highlight the ability of a phytochemical to induce the opposite effect depending on the target tissue. Still considering glucuronide derivatives of quercetin, a mixture (2.5–10 μM) of Q-3-GR, Q-7-GR, and Q-4′-GR was shown to induce apoptosis and cell cycle arrest (S and G2/M phases) in human lung carcinoma cells (NCL-H209) [165]. The apoptotic pathway was shown to be mitochondrial-dependent, as Bax and Bak’s expression was increased, cyt c release was increased but Bcl-2 expression was reduced [165].

Concerning the overall activity of an extract, apigenin derivatives action cannot be excluded, however, a few studies have analyzed their ability to modulate cell death pathways. Apigenin-7-*O*-glucoside (A-7-G) induced both apoptosis and autophagy in human gastric adenocarcinoma cells (AGS), confirmed by increased cleavage of caspase-8 and PARP, for apoptosis, and increased expression of LC3-II, beclin-1 and p62 confirming autophagy [166]. The cell cycle of AGS cells exposed to A-7-G was arrested in the G2/M phase, as the flavonoids modulated the expression of cyclin B1, M-phase inducer phosphatase 3 (CDC25C), and cyclin-dependent kinase 1 (CDK1). Reduced phosphorylation of Akt and PI3K was also observed [166].

#### 4.2.3. Pentacyclic Triterpenoids

Among the pentacyclic triterpenoids identified in *Thymus* spp. extracts, OA, and UA have been shown to be present in various species. These compounds have been thoroughly described in various cell line models, being highly effective anti-tumoral agents, as will be discussed below. In Table 5 we present various studies reporting OA and UA’s ability to induce cell death.

**Table 5:** Cell death modulation by oleanolic and ursolic acids in in vitro and in vivo animal experimental models.

Terpenoid	Concentration	Experimental Model	Observations	Ref.
Oleanolic acid	87.6–131.3 µM	Human pancreatic adenocarcinoma cells (Panc-28)	Induced apoptosis Cell cycle arrest (S and G2/M phases) Downregulation of p21, survivin, Bcl-2 Induced depolarization of mitochondrial membrane potential, cyt C release, PARP cleavage, caspase-3 and-9 activation	[175]
	4 and 8 µM	Human hepatic carcinoma cells (Huh7)	Induced apoptosis DNA fragmentation Induced depolarization of mitochondrial membrane potential Reduced Na^+^/K^+^-ATPase activity Increased caspase-3 and -8 activity	[176]
		Human hepatic carcinoma cells (Hep3B)		
		Human hepatic carcinoma cells (HepG2)		
		Human hepatic carcinoma cells (HA22T)		
	40 µM	Human hepatic carcinoma cells (HepG2)	Induced apoptosis Increased Bax expression, cyt c release, and cleavage of PARP Increased caspase-3 and -9 activity Reduced Bcl-2, p-Akt, and p-mTOR expression Cell cycle arrest in G2/M	[177]
	219 µM	Human lung carcinoma cells (A549)	Induced apoptosis Increased p-38 MAPK, p-JNK, and p-ERK Increased cyt c release and cleavage of PARP and caspase-3 and -9 Promoted mitochondrial translocation of Bax and Bim	[178]
	219 µM	Human pancreas adenocarcinoma cells (BXPC3)	Induced apoptosis Increased p-38 MAPK, p-JNK expression Increased cyt c release and cleavage of PARP and caspase-3 and -9 Promoted mitochondrial translocation of Bax and Bim	
	20 µM	Human hepatic carcinoma cells (Huh7)	Induced apoptosis Induced depolarization of mitochondrial membrane potential Increased Bax expression and cyt c release Reduced Bcl-2 expression	[179]
	80 µM	Human myeloid leukemia cells (HL60)	Induced apoptosis Cell cycle arrest (G1 phase) Increased caspase-3 and PARP cleavage	[180]
	30 and 60 µM	Human hepatic carcinoma cells (SMMC-7721)	Induced apoptosis and autophagy Induced depolarization of mitochondrial membrane potential Increased Bax, Beclin, and LC3 expression Reduced p-mTOR, p-Akt, Bcl-2, and p62 expression	[181]
	36 µM	Human papillomavirus-related endocervical adenocarcinoma cells (SGC-7901) *	Induced autophagy Increase in p-AMK, Beclin-1, and LC3-II expression Decreased p-PI3K, p-Akt, p-ERK1/2, p-p38 and p-mTOR expression	[182]
	36 µM	Human gastric mucinous adenocarcinoma cells (MGC-803)		
	36 µM	Human papillomavirus-related endocervical adenocarcinoma cells (BGC-823) *		
Ursolic acid	20 µM	Human hepatic carcinoma cells (Huh7)	Induced apoptosis Induced depolarization of mitochondrial membrane potential Increased Bax expression, cyt c release, cleavage of PARP, and caspase-3 and -9 activity Reduced Bcl-2 expression	[179]
	4 and 8 µM	Human hepatic carcinoma cells (Huh7)	Induced apoptosis DNA fragmentation Induced depolarization of mitochondrial membrane potential Reduced Na+/K+-ATPase activity Increased caspase-3 and -8 activity	[176]
		Human hepatic carcinoma cells (Hep3B)		
		Human hepatic carcinoma cells (HepG2)		
		Human hepatic carcinoma cells (HA22T)		
	10–30 µM	Mouse lymphoblast hybridoma (TC-1)	Induce autophagy Increased LC3-II and Autophagy related 5 (Atg5) expression	[183]
	30 µM	Human cervix adenocarcinoma (HeLa)	Induce apoptosis DNA damage	
	40 µM	Human glioblastoma cells (U87MG)	Cell cycle arrest (G1 phase) Decreased expression of cyclin D1, D3 and E, and cyclin-dependent kinase 4 (CDK4) Induced autophagy Increased LC3-II expression, p21 and p27 Modulation of CaMKK-AMPK-mTOR kinase pathway	[184]
	20 µM	Human lung carcinoma cells (A549)	Induced apoptosis Cell cycle arrest (G1 phase) Increased p53, p21/WAF1, Fas/APO-1, Fas and Bax expression Decreased expression of Bcl-2, Bcl-Xl cyclin D1, D2 and E, CDK2, CDK4 and CDK6 Inhibition of NF-kB activity	[185]
	53 µM	Human breast adenocarcinoma cells (MCF-7)	Induced apoptosis Induced PARP cleavage Downregulation of Bcl-2	[186]

*—cell lines with classification issue; Referred to as classified in Cellosaurus database.

As seen for both phenolic acids (Table 3) and flavonoid derivatives (Table 4), hepatic cancer cells are frequently used to evaluate phytochemicals’ anti-proliferative activity and are also susceptible to the terpenoids OA and UA action. The hepatic carcinoma cell lines Huh7, Hep3B, HepG2, and HA22T initiated apoptosis when exposed to 4–8 µM of either OA or UA, confirmed by various markers such as DNA fragmentation, mitochondrial membrane potential depolarization, and increased caspases activity [176]. A different study reported that HepG2 cells exposed to 40 µM OA presented markers of intrinsic apoptosis, with cyt c released to the cytoplasm, increased Bax expression, as well as caspase-3 and -9 activity and cleaved PARP [177]. HepG2 cells presented cell cycle arrest in G2/M and reduced phosphorylation of Akt [177]. Similar findings were reported for Huh7 cells exposed to 20 µM OA or UA, with activation of the mitochondria-dependent pathway, but without cell cycle arrest [179]. In SMMC-7721 (human hepatic carcinoma cells), in addition to apoptosis, exposure to 30–60 µM OA increased beclin-1 and LC3 expression, indicating autophagy, and reduced p-Akt and p-mTOR expression [181]. The same pattern, with reduced p-Akt and p-mTOR and upregulation of beclin-1 and LC3-II, was observed in human papilloma virus-related endocervical adenocarcinoma cells (SGC-7901 and BGC-823) and in human gastric mucinous adenocarcinoma cells (MGC-803) exposed to 36 µM OA, thus autophagy being indicated as the process involved in cell death [182]. In addition, OA induced lesser cytotoxicity to gastric normal cell lines GES-1 (human) and RGM-1 (rat) [182]. UA is also able to induce autophagy, as observed for mouse lymphoblast hybridoma (TC-1) [183] and human glioblastoma cells (U87MG) [184], being also capable of inducing apoptosis in a wide variety of cancer cell types such as breast [186], cervix [183], or lung [185]. In this last, A549 cells exposed to 20 µM OA, showed cell death through increased expression of p53, p21/WAF1, Fas/APO-1, Fas, and Bax, and cell cycle arrest, with marked decreased expression of cyclins D1, D2, and E, as well as CDK2, CDK4 and CDK6 [185].

**Figure 6:**
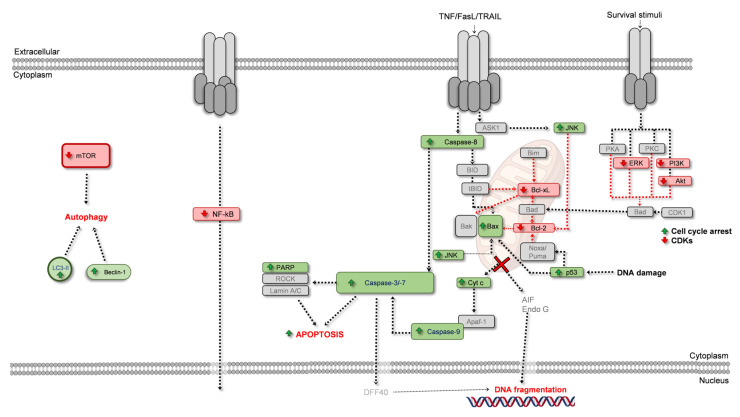
Main proteins and cellular pathways involved in phytochemicals’ anti-tumoral activity. Downregulation (red arrows) and upregulation (green arrows).

A relevant remark should be made on the OA effect in pancreas cancer cell models. In human pancreas adenocarcinoma cells (BXPC3), OA (219 µM) induced intrinsic-pathway apoptosis with the involvement of Bax and Bim, and cyt c release, leading to caspase-3 and -9 activation and PARP cleavage [178]. In addition, in Panc-28 (human pancreas adenocarcinoma) [175], OA induced downregulation of p21, Bcl-2, and also of surviving. Survivin is an anti-apoptotic protein, part of cell division in normal cells, but its high expression is associated with poor outcomes in cancer patients [187]. Nevertheless, despite the overall focus on the cytotoxicity of these compounds, in vivo studies proved that OA can also induce protective activity, such as Nrf2 activation to prevent liver toxicity induced by acetaminophen [188]. In Figure 6, the major upregulations and downregulations induced by phytochemicals’ anti-tumoral activity are illustrated.

As stated above, betulinic and corosolic acids were also identified in *Thymus* spp. extracts [95]. It was reported that betulinic acid can induce apoptosis and autophagy in various cancer cell lines [189,190,191,192,193,194]. Corosolic acid is no exception to the various reports of modulation of apoptosis and autophagy pathways by pentacyclic triterpenoids [195,196,197,198,199]. As discussed above, it cannot be excluded that future analysis of the phytochemical profile of already characterized species may reveal new phytochemicals or the presence of so-far not detected compounds. Either through the constant development of analytical methods to deepen our knowledge of the phytochemical profile, the wide range of extraction methods that are not homogeneous among authors, or the future analysis of plants grown/harvested in different conditions can produce significantly different outcomes. As seen in Table 2, Table 3 and Table 4, phenolic acids, glycoside derivatives of flavonoids and triterpenoids present the capacity to modulate cell survival and cell death pathways.

The reports suggest that the dose and the experimental model influence the results. For example, for CA (Table 2), protective effects were observed at 400 µM, but concentrations of 100, 200, or 300 µM also induced apoptosis in various cell lines. In Table 2 and Table 3, it is also possible to observe that 50 µM of SAA induced both anti-apoptotic and pro-apoptotic effects depending on the cell line. Thus, it is hard to predict the effect of an individual phytochemical. This problem scales up when considering an extract, a complex mixture of phytochemicals. The variation of an extract’s concentration in a specific phytochemical may also represent a variation in the concentration of several phytochemicals that compose the extract. Additionally, synergisms between compounds also affect the overall activity. For example, it is known that apigenin and luteolin increase RA’s bioavailability in the intestinal barrier [200]. At this time, the synergisms between the various components of an extract when in contact with a biological system are practically unknown. The effects can add up, and agonist and antagonist compounds may be found in the same extracts. Future studies focusing on the variability of complex matrices such as extracts are mandatory. The interactions of the various components may allow us to produce improved pharmacological formulations, taking advantage of the knowledge acquired from phytochemicals synergisms and antagonisms, with targeted approaches to cell survival or cell death mechanisms.

In addition, it is relevant to highlight that most studies reporting the anti-tumoral properties of both extracts and their phytochemicals, use only tumoral cell lines. It is critical that new studies focusing on the role of these natural products using both normal and tumoral cell lines, originating from the same tissue, be carried out and made available. These will provide information regarding the modulation of target proteins involved in apoptosis and/or necrosis in healthy conditions and also will contribute to evaluating the safety profile. These should be followed by a scale-up in the experimental models, through co-culture of non-tumoral/tumoral cell lines, 3D cell culture (e.g., organoids), up to complete in vivo studies that consider the administration, metabolization, bioavailability, and ability to target tumoral cells and protect normal cells, especially when used as adjuvants of chemotherapy drugs.

## 5. Conclusions

*Thymus* spp. extracts present various phenolic acids, flavonoids, and terpenoids with high pharmaceutical potential. Among them, caffeic, rosmarinic, salvianolic, oleanolic, and ursolic acids, as well as glycoside derivatives of flavonoids present the ability to modulate cell death signaling pathways. Interestingly this modulation is similar to what is reported for whole thyme extracts. Both the extracts and their main components reveal the high potential for new treatment approaches aiming at targeted cellular protection or cell death, both as effectors or adjuvants. This review also highlighted the significance of these phytochemicals in the overall bioactivity of a complex matrix. The apoptotic pathway is by far the most addressed process regarding cell death induction/prevention, but reports of necroptosis and autophagy are also being addressed, even revealing that multiple processes can be triggered simultaneously. There is a need to upgrade the knowledge from the effect of single phytochemicals to mixtures of these compounds, detailing the synergisms and antagonism in motion. The knowledge is essential to understand the bioactivities of complex matrices such as *Thymus* spp. extracts, and to better formulate new pharmaceutical formulations based on these natural products.

## Figures and Tables

**Table 1:** Summary of common phytochemicals identified in some *Thymus* spp. extracts considering phenolic acids, *O*-linked glycoside derivatives of flavonoids, and pentacyclic triterpenoids.

*Thymus* spp.	Main Phytochemicals											Ref.
	Phenolic Acids			Flavonoid Derivatives						Terpenoids		
	CA	RA	SA	E	A	L	Q	C	N	OA	UA	
*Thymus pulegioides*	•	•	SAI	-di-*O*-H -*O*-H -*O*-Hrn	-*O*-Hrn	-*O*-H -*O*-Hrn	-	-*O*-H	-*O*-H	•	•	[77,95]
*Thymus. fragrantissimus*	-	•	SAK SAI	-*O*-Hrn	-*O*-Hrn	-*O*-H -*O*-Hrn	r.	r.	r.	-	-	[73]
*Thymus carnosus*	•	•	SAA iso SAA SAK SAI	-*O*-H	r.	-*O*-H -*O*-H-P	r.	r.	-	•	•	[78]
*Thymus mastichina*	•	•	SAA iso SAE/B SAI	r.	r.	-*O*-H	-*O*-H	-*O*-Hrn	r.	•	•	[71,96]
*Thymus zygis*	•	•	SAK SAI	-*O*-H	r.	-*O*-H -*O*-Hrn	-*O*-H -O-A-H	r.	r.	•	•	[72]
*Thymus vulgaris*	•	•	SAA iso SAK SAI	-*O*-H	r.	-*O*-H -*O*-Hrn	-	r.	r.	•	•	[59,86,87]
*Thymus sibthorpii*	•	•	-	-	-*O*-H	-*O*-H -*O*-Rut	-	-	-	•	•	[95]
*Thymus serpyllum*	•	•	SAK SAI	-	-*O*-H	-*O*-H -*O*-Rut	-	-	-	•	•	[95,97]
*Thymus praecox*	•	•	-	-	-*O*-H	-*O*-H -*O*-Rut	-	-	-	•	•	[95]
*Thymus austriacus*	•	•	-	-	r.	-*O*-Rut	-	-	-	•	•	[95]
*Thymus × oblongifolius*	•	•	-	-	-	-*O*-Rut	-	-	-	•	•	[95]
*Thymus longicaulis*	•	•	SAA iso SAK SAK iso	-	-	-*O*-H -O-P -*O*-Hrn -*O*-Rut	-*O*-H	-	-	•	•	[94,95]
*Thymus algeriensis*	•	• -*O*-H	SAA SAK SAK iso SAE iso SAB	-*O*-H	-*O*-H -*O*-Hrn	-*O*-H -*O*-Hrn	-*O*-H	-	r.	-	•	[98,99,100,101]
*Thymus fontanesii*	-	•	SAA SAA iso SAK	-*O*-H	*O*-Hrn	-*O*-Hrn	*O*-Hrn	-	-	-	-	[99,102]
*Thymus munbyanus*	-	•	SAA	-*O*-H	-	-*O*-H -*O*-Hrn	-*O*-Hrn	-	-	•	•	[103]
*Thymus serrulatus*	-	•	SAA SAB SAF SAK iso SAK iso	-*O*-H	r.	-*O*-H -*O*-Hrn	-*O*-H -*O*-Hrn	-	-	-	-	[104]
*Thymus* × *citriodorus*	•	•	SAK SAI	-*O*-H	r.	-*O*-H -*O*-Hrn	r.	-*O*-H	r.	•	•	[59,84,86]

Notes: •: present; -: not present; r.: residual content/low content of various derivatives; CA: caffeic acid; RA: rosmarinic acid; SA: salvianolic acids; SAA: salvianolic acid A; SAB: salvianolic acid B; SAE: salvianolic acid E; SAF: salvianolic acid F; SAI: salvianolic acid I; SAK: salvianolic acid K; E: Eriodictyol; A: apigenin; L: luteolin; Q: quercetin; C: chrysoeriol; N: naringenin; OA: oleanolic acid; UA: ursolic acid; Derivatives -*O-*H: *O*-hexoside; -*O*-P: -*O*-pentoside; -*O-*Hrn: -*O*-hexuronide; -*O*-A-H: *O*-acetyl-hexoside; -*O-*H-P: *O*-hexoside-pentoside; -*O*-Rut: *O*-rutinoside; iso: isomer. Blue text: phenolic acids; green text: flavonoids; dark orange text: pentacyclic triterpenoids.

## Data Availability

Not applicable.
